# The Clinical Phenotype of Green Pit Viper (*Trimeresurus* spp.) Envenomation: A Comprehensive Review

**DOI:** 10.3390/jcm15218569

**Published:** 2026-11-03

**Authors:** Giovanni Paolino, Matteo Riccardo Di Nicola, Rachele Bardelli, Noemi Brigenti, Nicola Zerbinati, Santo Raffaele Mercuri, Andrea Carugno

**Affiliations:** 1 Dermatology Unit, Università Vita-Salute San Raffaele, 20132 Milan, Italy; paolino.giovanni@hsr.it (G.P.); mercuri.santoraffaele@hsr.it (S.R.M.); 2 Dermatology Clinic, IRCCS San Raffaele Scientific Institute, 20132 Milan, Italy; 3 Istituto Zooprofilattico Sperimentale del Piemonte, Liguria e Valle d’Aosta, 10154 Turin, Italy; matteoriccardo.dinicola@izsplv.it; 4 Dermatology Unit, Ospedale di Circolo e Fondazione Macchi, ASST Sette Laghi, 21100 Varese, Italy; rachele.bardelli@asst-settelaghi.it (R.B.); nicola.zerbinati@uninsubria.it (N.Z.); 5 Section of Dermatology and Venereology, Department of Medicine, University of Verona, 37126 Verona, Italy; noemi.brigenti@studenti.univr.it; 6 Department of Medicine and Innovation Technology (DiMIT), University of Insubria, 21100 Varese, Italy; 7 Department of Medicine and Surgery, University of Insubria, 21100 Varese, Italy

**Keywords:** green pit viper, *Trimeresurus*, snakebite envenomation, coagulopathy, antivenom, species identification, Southeast Asia

## Abstract

Green pit vipers of the genus *Trimeresurus* are among the leading causes of snakebite envenomation across tropical and subtropical Asia, yet their clinical phenotype has been less systematically characterized than that of other medically important Asian viperids. This comprehensive review synthesizes the published clinical literature on human *Trimeresurus* envenomation, drawing on 70 clinical reports encompassing 4720 reported patient episodes, and integrates these data with current taxonomic and venomic evidence. Envenomations cluster in East, Southeast, and South Asia. The single most important limitation of this literature is the unreliability of species attribution: 55% of reported cases are attributed only at genus level or derive from cohorts in which two or more *Trimeresurus* species are reported together and cannot be separated, a figure consistent with the 56% of snakebites left unidentified in a recent 40,817-patient Chinese survey. Bites involve the extremities almost exclusively, with a predominance of the lower limb (55% versus 45%). The clinical picture is dominated by local effects (i.e., extensive edema and pain in the large majority of patients, secondary infection in approximately one in six, and cutaneous necrosis in approximately one in thirty) and by systemic hemotoxicity driven by venom-induced consumptive coagulopathy. Renal involvement is uncommon, in contrast to *Daboia russelii* envenomation, and severe neurotoxicity is exceptional. Documented mortality in the reviewed hospital-based literature is low (two deaths among 3387 patients in reports documenting outcome, <0.1%) but is not zero, and hospital-based series cannot capture pre-hospital deaths. Management centers on conservative wound care, serial coagulation monitoring, and selective antivenom administration; routine fasciotomy is not justified without objective evidence of compartment syndrome. Reliable species identification, standardized clinical reporting, and long-term functional follow-up remain the principal unmet needs.

## 1. Introduction

Snakebite envenomation is a major and persistently neglected public health problem; in 2017 the World Health Organization formally classified snakebite envenoming as a neglected tropical disease, highlighting the urgent need for improved epidemiological surveillance, clinical management, and access to effective antivenoms [1]. While the global burden of snakebite has been extensively documented in regions such as sub-Saharan Africa and South Asia, important gaps persist regarding the clinical spectrum and real-world management of many medically relevant snake genera, such as *Trimeresurus* spp.

Among Asian venomous snakes, species belonging to the genus *Trimeresurus* (green pit vipers) represent a major cause of human envenomation in East and Southeast Asia. These arboreal crotaline snakes are widely distributed in countries such as Thailand, China, Taiwan, Vietnam, and Malaysia, where human/snake encounters are facilitated by ecological overlap and expanding anthropogenic environments. Despite their medical relevance, *Trimeresurus* bites have historically received less global attention than envenomations caused by other viperid genera, partly because of their generally lower reported mortality, although the bites of this genus can induce local and systemic symptoms [2,3].

Clinically, *Trimeresurus* envenomation is characterized by prominent local effects(i.e., pain, progressive edema, and soft-tissue injury) together with a systemic hemostatic syndrome dominated by venom-induced consumptive coagulopathy. Local and soft-tissue manifestations are usually the earliest findings and the proximate reason for medical consultation [2,4], yet they remain incompletely quantified across the literature, and the reported frequency of systemic complications, including coagulopathy, renal involvement, and neuro-ocular symptoms, varies substantially between studies.

Another important challenge is the marked heterogeneity of species identification and clinical reporting. Many studies group cases under the broad label “green pit viper”, and denominators for specific clinical features are frequently inconsistent or absent [2,3,4]. This variability complicates cross-study comparison, limits risk stratification, and impedes the development of standardized management algorithms, particularly regarding antivenom indications. The difficulty is compounded by the taxonomic complexity of the genus itself, which has undergone repeated revision [5].

The aim of the present review is to comprehensively analyze published clinical data on *Trimeresurus* envenomation, focusing on epidemiological distribution, species involvement, cutaneous and systemic manifestations, and management patterns and outcomes. By providing an updated and structured synthesis of the available evidence, we sought to better define the clinical spectrum of *Trimeresurus* envenomation and to identify current gaps that may inform future research and clinical practice.

## 2. Evidence Base and Literature Search

The clinical evidence discussed in this review was identified through a structured search of PubMed, Embase, and the Cochrane CENTRAL database, from inception to 14 October 2026, combining the genus name *Trimeresurus* and its synonyms with the vernacular names of the recognized species and with terms for envenomation, snakebite, and venom. The complete search strings for all three databases, with the records retrieved by each, are reported in the Supplementary Materials (Table S1). Titles and abstracts were screened independently by two authors (G.P. and A.C.), and full texts of all potentially eligible reports were assessed by the same two authors, with disagreements resolved by discussion.

Clinical studies, retrospective and prospective observational cohorts, case series, and single case reports describing human envenomations attributed exclusively to members of the genus *Trimeresurus* were considered across the geographic range of the genus. Reports based solely on animal models, or on in vitro venom and proteomic characterization without human clinical data, were not used as clinical sources, nor were envenomations clearly attributable to other sympatric Asian viperid genera (such as *Daboia*, *Calloselasma*, *Protobothrops*, or *Deinagkistrodon*). Experimental, proteomic, and taxonomic studies are nonetheless cited throughout the review wherever they inform pathophysiology or nomenclature.

Because the genus has been subject to repeated nomenclatural revision, we applied a single taxonomic convention throughout: all names follow the Reptile Database as accessed on 13 October 2026, historical names are reported as published on first mention and then converted to the currently accepted combination, and no case is reassigned between genera on our own authority. A synonymy table mapping every name used in the included literature onto its current combination is provided as Table S4.

Applying this convention required an explicit rule for cohorts spanning more than one genus. Several large series from Taiwan report *Trimeresurus stejnegeri* together with *Protobothrops mucrosquamatus*, two taxa that are sympatric, that are covered by the same bivalent antivenom, and that were until recently placed in the same genus. Where the source permits the *Trimeresurus* cases to be identified, only those are retained; where it does not, the cohort is excluded from the clinical frequencies and reported separately, because its data cannot be attributed to either genus. The same rule was applied to series reporting *Trimeresurus* alongside *Tropidolaemus*, *Calloselasma*, or *Ovophis*. The consequences are quantified in Section 4.4.

The searches retrieved 856 records from PubMed, 938 from Embase, and 22 from the Cochrane CENTRAL register, giving 1816 database records in total. After removal of 762 duplicates, overwhelmingly Embase records already retrieved through PubMed, 1054 unique database records remained; 11 further reports were identified by other methods, six through citation searching of the included reports and five by reviewers during peer review, giving 1065 records for screening. No language restriction was applied at any stage. Forty-three of the screened records were published in a language other than English (29 Japanese, 8 Chinese, 3 German, and one each in French, Thai and Slovak); all were indexed with an English title, 10 also carried an English abstract, and 5 are among the studies included here. Of the 1065 records screened, 929 were excluded and 136 full texts were assessed. Sixty-six were not retained: reviews, epidemiological summaries, or toxin-focused studies without original patient data (*n* = 27), among them a review of the ocular sequelae of snakebite envenoming [6] that had previously been treated as a primary source; conference abstracts and preprints, which do not permit extraction of the data tabulated here and in several instances duplicate a full publication already included (*n* = 13); animal or in vitro studies (*n* = 8); studies in which taxonomic reclassification placed the offending species outside the genus of interest (n = 6); full texts that could not be accessed (*n* = 4); veterinary studies (*n* = 2); duplicate or nested cohorts from the same center (*n* = 2); cohorts pooling *Trimeresurus* and *Protobothrops* without disaggregation (*n* = 2); a report concerning a Neotropical species also known vernacularly as a green pit viper (*n* = 1); and a study of behavioral ecology (n = 1). Seventy studies were included. Sixty-two of them contribute to the pooled clinical frequencies; of the remaining eight, three are cited only for the species counts they permit, two were retained in the bibliography as historical *Trimeresurus* reports but excluded from the counts after reassignment to *Craspedocephalus*, two are cited only for specific findings, and one contributes a fatality but no extractable denominator. Each is identified individually in Table S2. The selection process is summarized in Figure S1.

Seventy clinical reports [2,3,4,7,8,9,10,11,12,13,14,15,16,17,18,19,20,21,22,23,24,25,26,27,28,29,30,31,32,33,34,35,36,37,38,39,40,41,42,43,44,45,46,47,48,49,50,51,52,53,54,55,56,57,58,59,60,61,62,63,64,65,66,67,68,69,70,71,72,73] form the clinical basis of the sections that follow, comprising 4720 reported patient episodes attributed to *Trimeresurus* species and spanning 1973 to 2026, with a progressive increase in publications over the last decade. Most are case series, the remainder single case reports. Eight of the 70 do not contribute to the pooled clinical frequencies, for the reasons given below and recorded individually in Table S2.

Because reporting granularity varied widely across sources, the frequencies quoted throughout this review are descriptive summaries of what the published literature reports and not effect estimates derived from a formal meta-analysis: no quantitative pooling, weighting, or risk-of-bias appraisal was undertaken. They are crude, unweighted proportions, and no confidence intervals are given because no pooling was performed. Percentages are reported to the nearest whole number, which better reflects the precision the underlying data support.

Every frequency is expressed against an explicit, variable-specific denominator: the total number of patients in the reports that documented the item in question. Table 1 and Table 2 state this denominator and the number of contributing studies for every variable, and Table S2 records, study by study, which outcome domains each report documented. We also report, for comparison, the proportion obtained using a common denominator across all reports documenting at least one finding in the relevant domain. Both approaches are biased, in opposite directions. Variable-specific denominators inflate the apparent frequency of rare complications, because reports that looked for a rare finding are disproportionately those that found it. A common denominator avoids that inflation but enriches the denominator for studies that systematically recorded the domain in question, which may not be representative of practice.

For items documented by fewer than ten reports we therefore report the figure descriptively as “reported in k studies (n = N): x%”, because for these items the choice of denominator changes the result by up to an order of magnitude. Erythema illustrates the problem: three reports assessed it, and in those three it was present in roughly three-quarters of patients, so a pooled figure of 2% would reflect non-assessment, not absence.

Two further restrictions follow from the structure of the available data. Cohorts selected on the presence of a clinical feature are excluded from the frequency for that feature: the Hong Kong cohort of Chan et al. [60], for example, enrolled only patients who had developed at least grade 2 coagulopathy, and including it would yield a coagulopathy frequency of 100% by construction. Aggregate epidemiological surveys that report clinical findings for a whole national cohort rather than by species are cited for the species-specific data they do contain (case counts, severity distribution, and outcome) but do not contribute to the pooled clinical frequencies. A report available to us only as an abstract [37] describes local symptoms qualitatively, without patient counts; it contributes its *Trimeresurus* case count (n = 265) but no clinical frequency.

Finally, the figures below describe reported patient episodes rather than confirmed unique patients. Three of the included reports derive from the same emergency department in Bangkok with partly overlapping enrollment periods, and two Indian reports derive from the same community health center over consecutive periods. Patient-level data are not available to us, so exact de-duplication is not possible; where cohorts overlap we have retained the larger report, excluded a cohort whose enrollment window falls entirely inside another from the same center, and recorded every such decision in Table S2. Some patients may nonetheless be represented more than once, and the totals should be read accordingly.

For the purposes of this review, envenomation was classified as “local” when clinical manifestations were confined to the bite site and surrounding tissue (e.g., pain, edema, ecchymosis, and necrosis) without laboratory or clinical evidence of systemic involvement, and as “systemic” when hematologic (coagulopathy, bleeding), renal, neurologic, or cardiorespiratory abnormalities were documented, regardless of the severity of local findings. This classification was applied post hoc based on the clinical and laboratory data reported in each source study, given the absence of a standardized severity grading system across the included literature.

This article is therefore a comprehensive narrative synthesis rather than a systematic review: although the literature search was structured and is reproducible, the included studies were not formally appraised for methodological quality and their data were not meta-analyzed. The search string and the study selection diagram are provided as Supplementary Materials so that the evidence base underlying the figures reported below can be inspected in full.

## 3. Taxonomy and Biogeography of the Genus *Trimeresurus*

*Trimeresurus* Lacépède, 1804 is a species-rich genus of Asian pit vipers in the family Viperidae and subfamily Crotalinae, encompassing terrestrial to arboreal forms, many of which are predominantly nocturnal [74,75]. The group is morphologically heterogeneous, and close external similarity among related taxa, together with sexual dimorphism and geographic variation, has repeatedly obscured deeply differentiated lineages [74,76,77]. Integrative analyses combining morphological and molecular evidence have therefore prompted substantial revisions of species boundaries and nomenclature across several complexes [76,78,79]. Within the broad generic concept currently favored, *Trimeresurus* is generally divided into six subgenera (i.e., *Trimeresurus* sensu stricto, *Parias*, *Popeia*, *Himalayophis*, *Sinovipera* and *Viridovipera*) although some recent treatments include *Sinovipera* within *Viridovipera*, indicating that the subgeneric arrangement is not fully settled [74,77,78]. The Reptile Database listed 60 valid species of *Trimeresurus* as of 13 October 2026 [80]; that figure is provisional, since it still includes *Trimeresurus liqibini*, which has been synonymized with *Trimeresurus loong* [81], and new species continue to be described [77,82,83]. The genus extends across tropical and subtropical Asia, from the Himalayan region and southern China through mainland Southeast Asia to the Malay Archipelago, Timor and the Lesser Sunda Islands [74,77]. Recent taxonomic discoveries have been especially frequent in the Indo-Burma region and southwestern and southern China, while mountain systems, karst landscapes, river basins, mangroves and islands across the broader range support numerous geographically restricted lineages [76,77,78,82,83,84]. This biogeographic structure combines a limited number of broadly distributed taxa with many narrow-range endemics, making reliable species identification strongly dependent on locality and current nomenclature [78,79].

## 4. Epidemiology of *Trimeresurus* Envenomation

### 4.1. Geographic Distribution

The reports reviewed here describe where *Trimeresurus* envenomation has been published, which is not the same as where it occurs. Publication intensity, research infrastructure, access to hospital care, and the size of individual cohorts determine this distribution at least as much as the underlying incidence does, and the figures below should be read as a map of the published literature, not of true disease incidence. With that caveat, Thailand contributed the largest share of reported cases by a wide margin, followed by China, Vietnam, and Taiwan, with further contributions from Malaysia, Hong Kong, India, and Bangladesh.

Only sporadic cases were reported from Laos, Myanmar, Nepal, Sri Lanka, and Indonesia. A single case from the United States reflects a captive-collection bite sustained by a private keeper and is not an autochthonous exposure [27]. Two reports, accounting for 9 patients, did not state the country of the bite; the geographic denominator is therefore stated separately from the overall total.

The dominance of two countries in the published record is itself a limitation: because Thailand and Taiwan together contribute the majority of reported patients, the pooled clinical phenotype described in this review is substantially a Thai and Taiwanese phenotype, and extrapolation to India, China, Indonesia, and Malaysia (where the species involved differ) is not warranted.

Land-use and climatic change are plausibly altering the geography of human-snake contact in these regions. Modeling work incorporating climate, land-cover, and population change projects measurable shifts in the global distribution of snakebite incidence by 2050 [85], and the implications of environmental change for snakebite burden have been set out in detail [86]. For South Asia specifically, where national-scale geospatial risk mapping [87] and nationally representative mortality data [88] provide the baseline against which any change must be measured, distribution models predict shifts in venomous-snake hotspots and in snakebite risk across India under future climate scenarios [89], and an ecological analysis in the Republic of Korea has reported an association between deforestation and snakebite incidence [90]. Direct evidence linking agricultural expansion to *Trimeresurus* bites specifically is not available, and the proposition should be read as a hypothesis consistent with the broader literature rather than as a documented trend for this genus.

### 4.2. Species Involved and the Challenge of Identification

**Figure 1:**
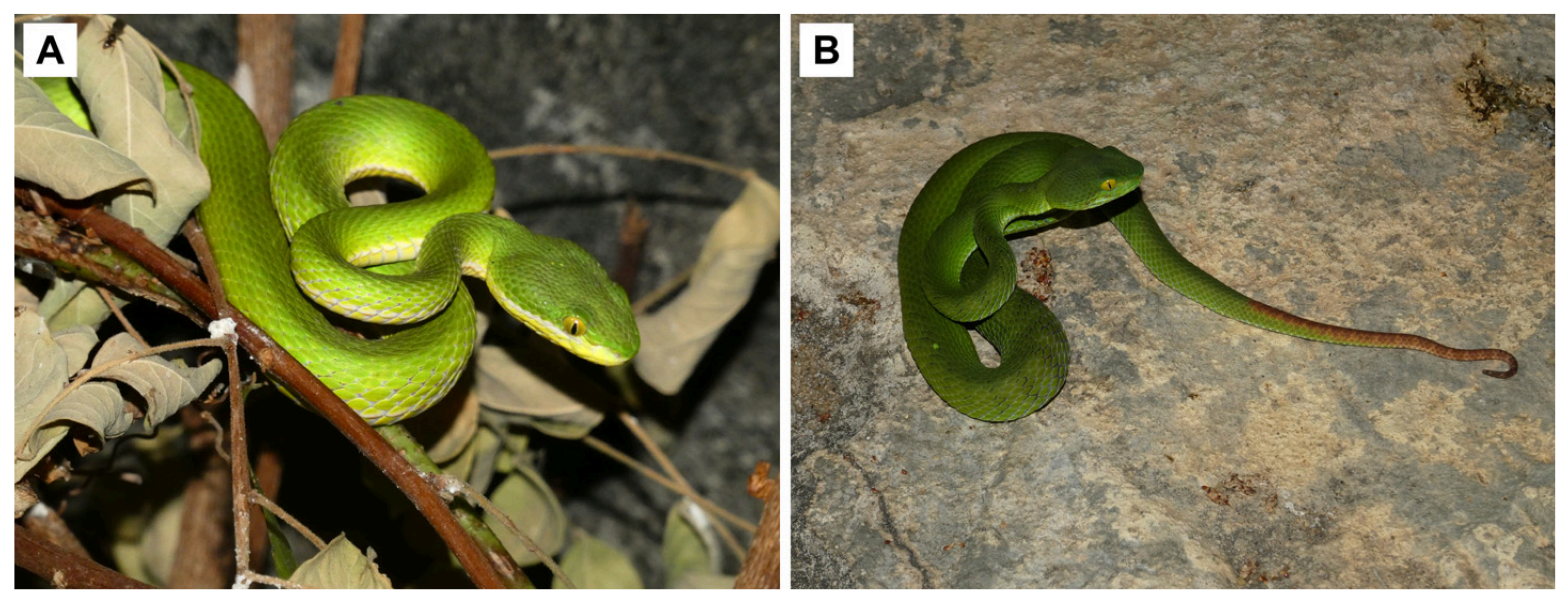
Field appearance of the two species that account for the largest single category in this review. (**A**) *Trimeresurus albolabris*; (**B**) *Trimeresurus macrops*. Cohorts reporting these two taxa together, without per-patient attribution, contribute 1375 of the 4720 cases reviewed here (29%). Photographs by G.P., Thailand, 2024.

Species attribution was heterogeneous across the included reports, and a majority of cases cannot be resolved to a single species. Cohorts reporting *Trimeresurus albolabris* together with *T. macrops* without disaggregation accounted for the largest single category (n = 1375; 29%). The second largest is not a species at all: 1086 cases (23%) are reported only as *Trimeresurus* spp. or “green pit viper”, exceeding the 1044 cases (22%) attributed to *T. albolabris* alone. *T. stejnegeri* accounted for 745 cases (16%), and a further 123 (3%) came from a cohort combining *T. albolabris* and *T. stejnegeri* (Table 1, Figure 1).

Among the remaining species, *T. purpureomaculatus* accounted for 149 cases (3%) and the closely related *T. erythrurus* and *T. salazar* for 93 (2%); a further 13 cases come from an early pediatric series in which *T. erythrurus* and *T. popeiorum* were reported together and cannot be separated [69]. All other taxa were individually uncommon, comprising *T. sabahi fucatus* (n = 26), *T. hageni* (n = 24), *T. sumatranus* (n = 15), *T. nebularis* (n = 13), *T. macrops* reported alone (n = 5), *T. sabahi sabahi* (n = 4), *T. gracilis* (n = 2), *T. venustus* (n = 1), one imported *T. flavomaculatus*, and one *T. insularis* [72]. Four taxa reported in the source literature under *Trimeresurus*, namely *T. malabaricus* [46], *T. trigonocephalus* [50], *T. borneensis*, and *T. wiroti*, are currently placed in *Craspedocephalus* [80] and were excluded on the same basis as *Protobothrops*; together they accounted for 36 cases.

*T. albolabris* is therefore the species most frequently reported as responsible for human envenomation in this literature, and recent cohorts from northeast India have drawn attention to the emerging clinical importance of *T. erythrurus* and *T. salazar*. Neither statement can be stronger than the identifications on which it rests, and the reliability of those identifications is examined in Section 4.4. The gap is clinically consequential, because intraspecific and interspecific venom variation, often driven by diet and geographic distribution, can alter both the clinical phenotype and the efficacy of regionally produced antivenoms [91,92]. Molecular diagnostics or rapid point-of-care venom detection could therefore prove decisive in shifting from syndromic management to targeted, species-specific therapy.

### 4.3. Demographic Profile, Bite Site, and Occupational Exposure

Among cases in which sex was reported, there was a male predominance (1559 of 2602 patients; 60%). Pediatric patients accounted for at least 525 cases, although age was not consistently reported. Four reports were dedicated to pediatric envenomation [35,36,41,69], one of which was a randomized controlled trial of prednisolone for limb edema in children [41], and the single best-documented neurological case in this literature was pediatric [48]. No included study compared pediatric and adult clinical courses directly, so any systematic difference between them remains unestablished.

Information on the anatomical site of the bite was available from 26 reports, in which 1628 bites were assigned to a specific site. Bites involved the extremities almost exclusively, with a predominance of the lower limb: 893 bites (55%) involved a lower extremity and 730 (45%) an upper extremity, while bites to the head or neck (n = 3) and trunk (n = 2) were exceptional. A further 114 patients came from reports that documented bite site for the cohort as a whole without a complete site-by-site breakdown and are excluded from this denominator. Where fang marks were explicitly recorded, they were documented in the majority of patients.

This distribution is consistent with a mixture of occupational and accidental exposure (treading on a terrestrial or low-lying snake on the one hand, and hand injury during agricultural work, rubber tapping, or attempts to handle the animal on the other) but the anatomical distribution alone does not establish the mechanism of exposure, and we draw no inference about it. We note that the single series in which upper-limb bites clearly predominated is also the only one in which every case carried an expert species identification [43], and its authors explicitly contrast their finding with earlier Malaysian studies reporting lower-limb predominance; whether this reflects a genuine difference in exposure or in case selection cannot be determined from the published data. The implication for prevention is that footwear alone addresses at most half the exposure, and that hand protection and snake-handling behavior require separate attention. The socioeconomic consequences of upper-limb injury in manual laborers align with the World Health Organization’s recognition of snakebite as an occupational disease of poverty, since prolonged hand disability translates directly into loss of livelihood [93].

### 4.4. Reliability of Species Attribution in the Source Literature

The preceding figures rest on species attributions made in the original reports, and the confidence those attributions can bear is limited. No included study used molecular confirmation of the offending snake. A minority relied on expert identification of a brought-in specimen or photograph, the Malaysian Remote Envenomation Consultancy Service series being the clearest example and the only one in which every case carries an expert determination [43]. The majority relied on clinician identification, syndromic inference, or hospital coding alone, and a substantial number reported no identification method at all. Table S2 records the method used, where stated, for every included report.

The consequences can be quantified. Of the 4720 cases with a recorded attribution, 2597 (55%) are attributed only at genus level or derive from cohorts in which two or more taxa are reported together and cannot be separated: 1086 are recorded simply as *Trimeresurus* spp. or “green pit viper”, 1375 come from cohorts reporting *T. albolabris* together with *T. macrops*, 123 from a cohort combining *T. albolabris* and *T. stejnegeri*, and 13 from an early pediatric series reporting *T. erythrurus* and *T. popeiorum* together. A further group of reports had to be set aside altogether because they pool *Trimeresurus* with *Protobothrops mucrosquamatus* without disaggregation [94,95]; the largest of these [95] ascertained cases by diagnostic code or by receipt of a bivalent antivenom that covers both genera. One further cohort proved disaggregable from a per-patient table and contributes only its five *Trimeresurus* cases [29]. External data corroborate this picture: a recent survey of 40,817 hospitalized snakebite patients across eleven Chinese provinces left 56% of snakes unidentified, with *T. stejnegeri* the most frequently identified pit viper at 11% [61].

Two further sources of uncertainty bear on the species-level conclusions. First, the ongoing revision of the *T. albolabris* complex [79] resolves it into multiple lineages whose ranges overlap the regions contributing most of the cases reviewed here, so an unquantifiable proportion of cases recorded as *T. albolabris* in the older literature probably represents other members of that complex; *T. macrops*, now recognized as distinct, is the clearest instance. Second, venom phenotype within this genus does not track phylogeny closely, with paralog composition and relative abundance varying within as well as among species [96]. Species-level clinical generalization is therefore weakened from two directions at once: the identifications are uncertain, and even a correct identification is an imperfect predictor of venom composition.

For these reasons the species-specific statements in this review should be read as clinical attributions rather than taxonomic determinations. This is the principal reason we place rapid, deployable species identification first among the research priorities in Section 10.

## 5. Venom Composition and Pathophysiological Basis

*Trimeresurus* venoms are complex protein mixtures in which a broadly conserved set of toxin families is expressed in species- and population-specific proportions [96,97,98]. Comparative LC-MS/MS consistently identifies snake venom metalloproteinases (SVMPs), snake venom serine proteases (SVSPs), phospholipases A2 (PLA_2_s), snake venom C-type lectin-related proteins (SV-CLRPs, commonly abbreviated snaclecs), disintegrins, and L-amino acid oxidases as the major components, with lower-abundance cysteine-rich secretory proteins, vascular endothelial growth factors, and hyaluronidases [96,97,98]. The families that matter most for the clinical phenotype described in Section 6 are the first three. SVMPs, present as P-I, P-II, and P-III classes in varying combinations, cleave extracellular-matrix and hemostatic substrates and are the principal determinants of microvascular injury, plasma extravasation, and local tissue damage [96,98,99]. SVSPs include fibrinogenolytic, thrombin-like, and factor-activating proteases that drive fibrinogen consumption and are the proximate cause of the defibrinogenation syndrome seen clinically [96,98,99,100,101,102]. PLA_2_ abundance is the most variable component: catalytically active Asp49 isoforms contribute inflammatory, cytolytic, and anticoagulant activity, while catalytically impaired Lys49 homologues retain pronounced membrane-disrupting and myotoxic effects [96,98,103]. Snaclecs and disintegrins modify platelet adhesion and aggregation [98]. Quantitative proteomes illustrate the scale of interspecific variation: *T. popeiorum* venom comprises 106 proteins in 12 families with approximately 60% proteolytic enzymes, whereas *T. erythrurus* venom comprises 159 proteins in 14 families dominated by SVSPs and snaclecs, with abundant Lys49 PLA_2_ isoforms [99,103]; a comparable proteome is available for *T. nebularis*, a *Popeia* species restricted to the Cameron Highlands of Malaysia [104]. At genus level SVMPs and SVSPs are the most consistently expressed families, but paralog composition and relative abundance vary within as well as among species and do not track phylogenetic affinity or geographic origin closely, so lineage alone does not predict venom phenotype [96] (a decoupling of venom composition from phylogeny also documented among viperids outside Asia [105]). Conspicuous geographic color polymorphism in *T. insularis* is not accompanied by corresponding proteomic divergence [106], a reminder that visual appearance is an unreliable guide to venom phenotype. Coverage remains uneven, with many recognized species lacking quantitative proteomes and several datasets based on pooled venom or few specimens [96,106].

One discordance deserves explicit comment, because it bears on the interpretation of the local phenotype. The low frequency of cutaneous necrosis described in Section 6.1 is often attributed to a low content of myotoxic PLA_2_s, yet *T. erythrurus* venom is rich in Lys49 PLA_2_ isoforms [103] while the clinical series available for that species report severe local swelling with comparatively infrequent necrosis [33,49,52]. Three considerations may be relevant: Lys49 homologues are catalytically inactive and act principally on skeletal muscle membranes, so myotoxic potential is not equivalent to dermonecrotic potential; relative abundance in a pooled-venom proteome is not a measure of specific activity in vivo; and the clinical definition of necrosis is not standardized across the reports reviewed here. These considerations do not resolve the discordance, and the proteomic basis for the comparatively low frequency of dermonecrosis in this genus is not established. Establishing it would require paired venom-proteomic and clinical-outcome data from the same patient populations, which do not currently exist for any *Trimeresurus* species.

## 6. Clinical Phenotype of *Trimeresurus* Envenomation

Taken together, the reviewed literature depicts green pit viper bites as a clinically relevant envenomation dominated by local effects and by a hemotoxic systemic syndrome. True lethality is exceedingly low, as discussed in Section 8, yet the morbidity associated with local tissue damage and coagulopathy represents a substantial burden for healthcare systems in endemic regions.

### 6.1. Local Cutaneous and Soft-Tissue Manifestations

Documentation of local skin and soft-tissue findings was heterogeneous across the available reports, as several large cohorts did not record individual cutaneous items. The frequencies discussed below are therefore expressed against the variable-specific denominators defined above (Table 2).

Edema or swelling was the most frequently documented local sign, recorded in 1700 of the 2526 patients from reports documenting it (67%), and was reported across almost all species. Pain at the bite site was documented in 1047 of 1333 patients (79%). Local ecchymosis or hematoma was recorded in 376 of 1223 patients (31%), consistent with the vasculotoxic phenotype of the genus. Secondary local infection, discussed in detail in Section 7.2, was documented in 94 of 560 patients (17%). Cutaneous necrosis was recorded in 76 of 2189 patients (3%); where time to necrosis was reported, onset typically occurred over several days. The less consistently assessed findings are reported here in the form that reflects their evidence base: blistering lesions were documented in 6 reports (vesicles, n = 96 of 1444; 7%) and bullae in 10 reports (n = 64 of 457; 14%), and erythema was explicitly assessed in only 3 reports, in which it was present in 55 of 76 patients (72%), a figure that demonstrates why a pooled denominator would misrepresent non-assessment as absence.

Taken together, these proportions define *Trimeresurus* envenomation as primarily a locally acting syndrome in which edema and pain are near-universal, secondary infection is common, and frank tissue necrosis is comparatively infrequent, though its reported frequency varies several-fold between cohorts, from approximately 2% in Thai and Hong Kong series of *T. albolabris* to 7% in a Taiwanese series of 185 *T. stejnegeri* envenomations [62]. We cannot determine from the published data whether this range reflects genuine species or regional differences, differing thresholds for diagnosing necrosis, or differences in case ascertainment, and we therefore present it as a range alongside the pooled figure.

Reported necrosis frequencies in *Trimeresurus* series are lower than those reported for Neotropical *Bothrops* species or for the Malayan pit viper (*Calloselasma rhodostoma*) [107,108], but no study has compared these genera directly using a common severity definition, and the comparison rests on indirect evidence. The mechanistic explanation usually offered, a lower content of myotoxic PLA_2_s, is not supported by the available proteomic data and is discussed in Section 5.

The clinically consequential point is a different one. Severe local swelling may prompt surgical intervention, and fasciotomy may be performed unnecessarily because venom-induced limb tenseness is genuinely difficult to distinguish from true ischemic compartment syndrome. The available evidence indicates that routine fasciotomy is rarely justified in crotaline envenomation without objective evidence of raised intracompartmental pressure, and that premature surgery may worsen morbidity by creating open wounds prone to secondary infection [108,109]; an experimental porcine model found fasciotomy to increase the extent of myonecrosis [110]. This recommendation does not depend on the absolute frequency of necrosis, and it should not be read as dismissing the clinical difficulty of the decision. Where intracompartmental pressure monitoring is available it should be used; where it is not, serial assessment by an experienced clinician is the only alternative. We recognize that routine surgical decompression remains standard practice in some centers, and that changing it requires clinical training and resources in addition to published evidence.

One further observation from the recent literature is clinically actionable: in the series of 185 *T. stejnegeri* envenomations, independent predictors of wound necrosis were the application of cold packs as first aid, the formation of bullae or blisters, and wound infection [62]. Bulla formation preceding necrosis has a plausible mechanistic basis in SVMP activity at the dermal-epidermal junction, and the association with cold packs is a specific argument against a first-aid measure still in use.

### 6.2. Systemic Manifestations: Coagulopathy and Bleeding

Systemic involvement was likewise variably captured across clinical reports and observational cohorts. The frequencies below are therefore expressed against the variable-specific denominators defined above (Table 2).

Laboratory-confirmed coagulopathy, usually reported as venom-induced consumptive coagulopathy or disseminated intravascular coagulation, was the most frequently documented systemic abnormality, recorded in 1058 of the 3134 patients from reports documenting it (34%). Overt systemic bleeding, the most frequent systemic clinical manifestation, was documented in 242 patients (10% of 2485), and was reported principally after envenomation by *T. albolabris*, *T. stejnegeri*, *T. purpureomaculatus*, and *T. erythrurus*. All counts are at patient level; where a patient had more than one bleeding event, the patient is counted once. Renal involvement was documented in 17 patients across 9 reports, the largest historical cluster deriving from an early series on the kidney in tropical snakebite [47], and acute respiratory distress in 2 patients across 2 reports. These figures are too sparse to support pooled severity estimates and likely reflect incomplete structured capture in cohorts focused on local and hemotoxic outcomes rather than true absence.

Mechanistically, *Trimeresurus* venoms contain potent thrombin-like enzymes (TLEs) and serine proteases (SVSPs) that cleave fibrinogen, producing a rapid defibrinogenation syndrome [100,101,102]; systemic coagulopathy and bleeding therefore constitute the main life-threatening risk of these envenomations, even though they are less frequent than local effects. Unlike envenomation by sympatric species such as Russell’s viper (*Daboia russelii*), which is characterized by severe acute kidney injury, renal involvement in *Trimeresurus* envenomation is remarkably uncommon, and severe neurotoxicity or acute respiratory distress are exceedingly rare [111]. Green pit viper envenomation is thus overwhelmingly a consumptive and hemorrhagic syndrome, lacking the severe nephrotoxic and presynaptic neurotoxic components seen in other Asian vipers.

### 6.3. Atypical Presentations: Neurologic, Renal, Hematological, and Respiratory Involvement

Neurologic involvement was documented in 4 of the 415 patients in the two reports that recorded it. The entire evidence base consists of one cohort reporting neurotoxic signs in 3 of 414 patients without detailed neurological characterization [51] and one fully described pediatric case [48]; the mechanism is unknown. In that case, a child presenting with ptosis, exotropia, and respiratory distress failed to improve after cobra and Malayan pit viper antivenoms, and the neurological and respiratory abnormalities resolved only after green pit viper antivenom was given [48]. Severe neurological signs after a presumed green pit viper bite in Asia properly raise the suspicion of misidentification with a sympatric neurotoxic species, and the sequence of antivenom responses in this case is the strongest available argument against that explanation. It remains a single case. These observations do not establish a neurotoxic component of *Trimeresurus* venom; they indicate only that a neurological presentation should not be taken to exclude the diagnosis, and that prospective confirmation is required.

Hematological presentations beyond consumptive coagulopathy have now been reported twice. A patient bitten by a white-lipped pit viper developed thrombotic microangiopathy [45], and a woman in rural Nepal bitten by a presumed *Trimeresurus* sp. developed microangiopathic hemolytic anemia with thrombocytopenia and coagulopathy, diagnosed syndromically where peripheral blood smear was unavailable, and recovered completely with supportive care and blood products in the absence of any antivenom [63]. Two cases do not establish a frequency, but they are consistent with each other and suggest that microangiopathy is a recognizable if rare pattern after envenomation by this genus, worth considering when thrombocytopenia is accompanied by anemia.

## 7. Management

Therapeutic management was reported with variable granularity across the included studies, reflecting differences in clinical setting, local protocols, and completeness of data capture. Overall, treatment strategies were consistent with the predominantly hemotoxic and vasculotoxic profile of *Trimeresurus* envenomation and were largely centered on careful clinical observation, laboratory monitoring, supportive care, and selective use of antivenom.

### 7.1. Antivenom: Indications, Paraspecificity, and Adverse Reactions

Antivenom administration was documented in 1752 of the 3471 patients from reports recording it (50%). Where the indication was specified, the decision was generally driven by progressive local envenomation, clinically relevant bleeding, or laboratory evidence of coagulopathy. Several large series explicitly described a selective rather than routine strategy, reflecting the moderate course of many *Trimeresurus* envenomations and the need to weigh benefit against adverse reactions. The products used included both monovalent and polyvalent F(ab’)_2_ preparations, largely dependent on regional availability.

Thresholds: the source literature applies thresholds inherited from general viperid practice rather than derived from *Trimeresurus* data, and it is worth stating them explicitly together with that caveat. The World Health Organization criteria for hemostatic abnormality (i.e., spontaneous distant bleeding, an unclotted 20-min whole-blood clotting test (20WBCT), INR above 1.2 or prothrombin time 4–5 s longer than the laboratory control, and a platelet count below 100 × 10^9^/L) provide the lowest treatment threshold in use [112] and were applied in the Hong Kong cohort [60]. Thai centers have used venous clotting time and thrombin time [4,54], and a prospective study has now characterized the relationship between 20WBCT clotting state and fibrinogen concentration in green pit viper envenomation specifically, which is directly relevant where fibrinogen assay is unavailable [64]. In Taiwan, delayed initiation of antivenom has been defined as administration more than 6 h after the bite [65]. No included study prospectively validated any of these thresholds against a clinical outcome, and this remains a concrete gap.

Timing: two recent cohorts quantify the consequences of delay, and their findings are not identical. In Hong Kong, door-to-antivenom time was independently associated with the duration of venom-induced coagulopathy, with a median of 4.2 h in a cohort selected for coagulopathy [60]. In Taiwan, delayed initiation beyond 6 h was independently associated with wound infection (adjusted odds ratio 3.99), with infection rates rising across strata of increasing delay [65]. By contrast, in a third cohort early antivenom administered within 6 h was not associated with a lower incidence of bullae, necrosis, coagulopathy, or thrombocytopenia [62]. The weight of evidence favors early administration where antivenom is indicated, but the discordance between these cohorts should not be flattened: the outcomes measured differ, and none of the three was designed as a trial of timing.

Local effects: the question of whether antivenom benefits isolated local envenomation has been addressed directly, and the answer is largely negative. In a randomized, double-blind, placebo-controlled trial, antivenom accelerated the resolution of limb edema to a statistically significant but, in the authors’ own assessment, not clinically significant degree, produced no difference in pain reduction, and the authors recommended against general use for local effects [44]. We therefore do not recommend antivenom for pain or local tenseness in the absence of progressive swelling or systemic signs.

Adverse reactions: reactions were documented in 53 of 917 patients from reports recording them (6%), including isolated anaphylaxis and delayed serum sickness [32,34]; reported early-reaction rates range from low in Thai series using F(ab’)_2_ product [53] to a high incidence of anaphylactic shock in one Lao series [55]. Their occurrence reinforces the need for judicious patient selection, monitoring during infusion, and readiness to treat hypersensitivity. Antivenom skin testing, still performed in many Asian centers, is discouraged by WHO guidance and performs poorly as a screening test [65].

Paraspecificity: products raised against *T. albolabris* or *T. stejnegeri* are widely used outside the range of the species against which they were raised, and the evidence supporting this practice is preclinical. Thai monovalent *T. albolabris* antivenom has shown cross-reactivity against several Indonesian members of the *Trimeresurus* complex [92], and the Taiwanese bivalent product raised against *T. stejnegeri* and *P. mucrosquamatus* appeared effective against the local toxicity of *T. gracilis* envenomation in the only clinical observation bearing on the question (two patients) [66]. The evidence from the Indian subcontinent is more discouraging. Indian polyvalent antivenom, produced against the “big four” species, shows poor immunorecognition and weak neutralization of *T. erythrurus* venom, conferring protection in rescue-type experiments only at very high antivenom-to-venom ratios [113]; comparative work across several Indian green pit viper venoms reached a similar conclusion [92]. A purpose-built bivalent equine F(ab’)_2_ antivenom against *T. erythrurus* and *T. popeiorum* has now been developed and shows markedly superior binding and neutralization relative to polyvalent antivenom in preclinical models, including cross-regional efficacy [114]; it has not yet been evaluated clinically. The practical implication is that where *T. albolabris* is not the predominant local species, reliance on *T. albolabris*-raised product rests on an assumption tested preclinically and not clinically, and that for northeast India the available polyvalent product should not be assumed effective.

### 7.2. Wound Care, Secondary Infection, and the Question of Fasciotomy

Supportive care constituted a central component of management in most studies. Intravenous fluid administration, analgesic therapy, and, in selected cases, transfusion support for clinically significant bleeding were described whenever detailed treatment information was available.

Antibiotic therapy was reported particularly in cohorts in which secondary local infection, cellulitis, or abscess formation occurred. These infectious complications were most frequently associated with bites attributed to *T. stejnegeri*, *T. albolabris*, *T. erythrurus*, and to cases recorded only as green pit viper. Nevertheless, the indication for antibiotics was not standardized across studies, and in many cases their use appeared to be empiric or institution dependent.

Secondary local infection deserves particular scrutiny. Snakebite wounds are initially sterile, and the early inflammatory response reflects venom-induced tissue injury rather than infection, so that primary venom effects are frequently misdiagnosed as bacterial cellulitis. Bacterial inoculation from the snake’s oral flora, from the skin, or from pre-hospital wound interference can nonetheless occur, and secondary infection is well documented. Reported frequencies vary widely: 7% in a Bangkok cohort in which antibiotics were prescribed to only 12% of patients and every culture was negative, with hemorrhagic blebs the sole identified risk factor [115], against 26% overall in a Taiwanese *T. stejnegeri* cohort in which the rate rose from 21% in patients receiving antivenom within 2 h to 48% in those treated beyond 6 h [65]. Where organisms were cultured they were frequently polymicrobial and included *Morganella morganii*, *Enterobacter cloacae*, and *Enterococcus faecalis*, species against which amoxicillin-clavulanate and first-generation cephalosporins perform poorly [65]. Routine prophylactic antibiotics are therefore not indicated in the absence of clinical evidence of infection, a position consistent with WHO guidance on antimicrobial stewardship [112,116], but a low threshold for reassessment is warranted in delayed presentations and in patients with hemorrhagic blebs, and where infection is established, third-generation cephalosporins, quinolones, or piperacillin-tazobactam are the more appropriate empirical choices [65].

Local wound management, including immobilization of the affected limb, wound cleansing, and close clinical observation, was consistently described as first-line care. Surgical intervention was performed in 196 of the 1109 patients from reports documenting it (18%), of which 14 were fasciotomies; the remainder comprised wound debridement, split- or full-thickness skin grafting, local flap coverage, and, in two cases, digital amputation. We use “performed” rather than “required” deliberately: the retrospective literature cannot establish that every procedure was necessary, which is precisely the point at issue in Section 6.1.

### 7.3. Laboratory Monitoring and Observation

Several studies also emphasized the importance of serial laboratory monitoring, particularly coagulation parameters, during the first 24 to 72 h after envenomation. This was especially highlighted for *T. albolabris*, *T. stejnegeri*, and *T. erythrurus*, in which delayed coagulopathy has been described. However, because monitoring schedules and laboratory thresholds were highly heterogeneous across the included reports, a pooled quantitative analysis of monitoring strategies was not feasible.

## 8. Outcomes and Mortality

The duration of hospital stay varied considerably across the reported cohorts, typically ranging from a few days for mild cases requiring only observation, to over a week for patients requiring antivenom, surgical interventions, or management of severe local tissue damage.

Across the reports documenting outcome, two deaths were recorded. The better-characterized is a 71-year-old woman in a Taiwanese series of 185 *T. stejnegeri* envenomations who developed coagulopathy (INR 6.2, aPTT > 224 s) and died of continuing gastrointestinal bleeding and hemorrhagic shock 18 h after admission, having not received fresh frozen plasma in time; renal impairment was also present [62]. The second is a fatality in a Hong Kong series of 242 snakebite admissions attributed to a probable *T. albolabris* bite, with species identification qualified as probable rather than confirmed and no case-level detail available [23]. Only the first is therefore attributable to a confirmed *Trimeresurus* species.

Documented mortality in this literature is consequently low (below 0.1% of the patients in reports recording outcome) but it is not zero, and three considerations limit what the figure can be taken to mean. Most reports are hospital-based and cannot capture deaths occurring before medical contact. Species identification was incomplete in a substantial proportion of cases, as set out in Section 4.4. And severe cases are over-represented in case reports while mild cases are systematically under-reported, so the denominator is not a population denominator. The national Chinese survey offers a useful external comparison: overall in-hospital mortality across 40,817 snakebite admissions of all species was 0.06%, and among *T. stejnegeri* bites specifically 89% were graded mild and 0.7% severe on admission, with 85% discharged healed or improved, but 15% discontinued treatment, a form of attrition that hospital-based series in this review do not capture at all [61]. *Trimeresurus* envenomation is thus a condition of substantial morbidity and low documented in-hospital lethality. Severe outcomes (such as systemic bleeding, renal involvement, compartment syndrome, respiratory complications, microangiopathy) are rare but documented, and their rarity in pooled hospital series does not remove the need for observation, particularly in delayed presentations, vulnerable patients, and resource-limited settings.

Overall, the available evidence suggests that while most *Trimeresurus* envenomations follow a favorable course under appropriate medical management, careful clinical and laboratory surveillance remains essential to promptly identify and treat the subset of patients who develop significant systemic toxicity.

## 9. Limitations of the Available Evidence

Several limitations affect both the body of evidence reviewed here and the present synthesis. As a comprehensive narrative review, this work did not incorporate a formal appraisal of methodological quality or a quantitative meta-analysis; the frequencies presented are descriptive, are drawn from heterogeneous denominators, and should be interpreted accordingly. Reporting across the primary literature was itself markedly heterogeneous. Most available studies are retrospective case series. Although both monovalent and polyvalent F(ab’)_2_ antivenoms were used across the reviewed studies, heterogeneous outcome reporting precludes any stratified appraisal of clinical efficacy by antivenom type, an important gap given the ongoing debate on paraspecificity discussed above. The absence of a standardized severity grading system across studies prevents robust comparison of treatment thresholds, and long-term functional follow-up of bitten limbs is virtually non-existent in the literature, leaving the true extent of chronic disability unknown.

Five further limitations deserve explicit statement. Language: no language restriction was applied, and five of the included reports are published in Chinese, Thai or Slovak. Across the whole search, 43 records were in a language other than English; all were indexed with an English title and 10 with an English abstract, so none was excluded on grounds of language, and no such category appears in Table S3. What the English-language record does not contain could not, however, be recovered for these reports. A more material restriction lies upstream: clinical literature indexed only in national databases such as CNKI or ThaiJO was not searched at all, so for a genus whose burden is concentrated in China, Taiwan, Thailand, and Vietnam our claim to comprehensiveness is limited to the internationally indexed literature. Publication bias: case reports over-represent severe and unusual presentations, which inflates the apparent frequency of rare complications, while mild and conservatively managed cases rarely reach publication, which biases the mortality denominator; these two biases act in opposite directions and we cannot quantify either. Temporal change: the evidence spans 1973 to 2026, a period over which antivenom manufacture, critical care, and coagulation diagnostics changed substantially, and the majority of patients derive from reports published since 2015; we have not attempted temporal stratification because the denominators do not support it. Attribution and ascertainment: hospital diagnostic coding may misclassify at genus as well as species level, and even expert morphological identification is unreliable for taxa within the *T. albolabris* complex without molecular confirmation. Mixed-species cohorts: two of the included series report clinical outcomes for all venomous snakebites together rather than by species, and only part of each cohort is attributable to this genus. In a pediatric series from southern China [36], 35 of 69 children were bitten by *T. albolabris*, the remainder by *Naja atra*, *Daboia siamensis*, *Bungarus multicinctus*, *Protobothrops mucrosquamatus*, or a snake that was not identified; in a Vietnamese series [24], 22 of 41 patients were bitten by *T. albolabris*, the remainder by *Protobothrops* species, *Deinagkistrodon acutus*, or an undefined snake. The outcome counts of these two reports therefore enter the pooled frequencies as published and are not specific to *Trimeresurus*; both are identified as such in Table S2.

Door-to-antivenom time, long considered unextractable from retrospective records, has now been reported and analyzed in two cohorts [60,65] and is associated with both the duration of coagulopathy and the risk of wound infection. It remains reported in only a small minority of series and could not be pooled, but it is no longer an unmeasured variable and should be a required field in future reporting.

We also note that the figures presented describe reported patient episodes rather than verified unique patients, for the reasons given in Section 2. Finally, because the clinical distinction between local venom effects and true surgical or systemic emergencies is genuinely difficult, future prospective studies would benefit from direct input by clinicians experienced in managing envenomation by this genus. In Taiwan and southern China such experience necessarily extends to *Protobothrops mucrosquamatus*, which is sympatric with *T. stejnegeri* and covered by the same bivalent antivenom; the clinical overlap between the two genera is precisely what makes species-resolved reporting difficult in that setting, which is why *Protobothrops*-pooled cohorts could not contribute to the frequencies reported here.

## 10. Conclusions and Future Directions

Clinicians should expect local cytotoxic and inflammatory effects to dominate the clinical picture after *Trimeresurus* bites (i.e., edema and pain in the large majority of patients, secondary infection in a substantial minority), while hemostatic disturbance is the predominant systemic manifestation and the principal source of life-threatening risk. Serial coagulation monitoring is therefore essential. In the reports reviewed here this consisted of the 20-min whole-blood clotting test at presentation and at 6-hourly intervals where laboratory testing was unavailable, and otherwise of prothrombin time or INR, fibrinogen, and platelet count at presentation and at 6, 12, and 24 h, continued to 72 h in patients who had received antivenom or whose initial results were abnormal; these intervals reflect reported practice and have not been formally validated. Antivenom should be reserved for progressive local envenomation or systemic coagulopathy and is not indicated for pain or local tenseness alone. While necrosis is infrequent, clinicians should be cautious before operating for severe edema, since routine fasciotomy is not justified without objective evidence of compartment syndrome and may itself increase morbidity.

Three research priorities follow, in order. First, deployable species identification. Of the options available, lateral-flow venom detection remains a research tool with no licensed product for this genus; portable sequencing is feasible at referral centers but not at first contact; and photographic identification with expert teleconsultation is the only approach currently deployable at scale, the Malaysian Remote Envenomation Consultancy Service being a working example [43]. Given that 55% of reported cases in this literature cannot be resolved to a single species, this is the limiting constraint on everything else. Second, standardized reporting. A minimum dataset should comprise: species identification and the method by which it was made; time from bite to presentation and to antivenom; bite site; local findings with a defined severity grade; coagulation parameters with sampling times; antivenom product, dose, and repeat dosing; adverse reactions; surgical procedures with indication; and functional outcome at 30 days. Prospective multicenter registries applying such a dataset, with uniform denominators, would resolve most of the heterogeneity that currently limits comparison. Third, antivenom evidence. Comparative studies of monovalent versus polyvalent products reporting clinical rather than surrogate endpoints are needed to settle the paraspecificity question, and the newly developed bivalent product against *T. erythrurus* and *T. popeiorum* [114] requires clinical evaluation in northeast India, where polyvalent antivenom is demonstrably inadequate [113]. Alongside these, structured long-term follow-up of limb function after severe local envenomation would quantify a chronic disability burden that current hospital-based series leave almost entirely unmeasured.

## Figures and Tables

**Table 1:** Epidemiological, clinical, and management features of *Trimeresurus* envenomation as reported in the reviewed literature.

Domain	Variable	*n*	%	Denominator
Study population	Reported patient episodes	4720	-	70
	Case series (studies)	52	74	70
	Case reports (studies)	18	26	70
Demographics	Male	1559	60	2602
	Female	966	40	2417
Species attribution	*T. albolabris*/*T. macrops* (mixed, not disaggregable)	1375	29	4720
	*T. albolabris*	1044	22	4720
	*Trimeresurus* spp./“green pit viper” (genus only)	1086	23	4720
	*T. stejnegeri*	745	16	4720
	*T. purpureomaculatus*	149	3	4720
	*T. albolabris*/*T. stejnegeri* (mixed)	123	3	4720
	*T. erythrurus*/*T. salazar*	93	2	4720
	*T. erythrurus*/*T. popeiorum* (mixed)	13	<1	4720
	Other *Trimeresurus* species (10 taxa, each < 1%)	92	2	4720
Bite site	Lower extremity	893	55	1628
	Upper extremity	730	45	1628
	Head/neck/trunk	5	<1	1628
	Site reported but not specified	114	-	1742
Management	Antivenom administration	1752	50	3471
	Hospitalization	411	36	1155
	Surgical intervention (any)	196	18	1109
	Fasciotomy	14	3	491
Outcomes	Antivenom adverse reactions	53	6	917
	Death	2	<0.1	3387

Abbreviations: spp., species pluralis (multiple species). Note: n/N (%) format throughout. Percentages are crude, unweighted proportions calculated against variable-specific denominators (the number of patients in reports that explicitly documented each item) and are rounded to the nearest whole number. Figures are descriptive and are not derived from a meta-analysis; no confidence intervals are given because no pooling was performed. The contributing studies for each variable are listed in Table S2. The two deaths are not drawn from the same denominator: the first [62] occurred in a cohort counted here in full, whereas the second [23] is reported in an all-species series of 242 snakebite admissions from which the *Trimeresurus*-specific sample size could not be separated, and which therefore contributes to the numerator but not to the denominator. Counting those 242 admissions as well would give 2 of 3629; the proportion stays below 0.1% either way.

**Table 2:** Cutaneous and systemic manifestations of *Trimeresurus* envenomation reported in the reviewed literature.

Domain	Manifestation	n	%	Denominator	Studies
Cutaneous, local	Edema/swelling	1700	67	2526	36
	Pain	1047	79	1333	30
	Ecchymosis/hematoma	376	31	1223	15
	Secondary infection	94	17	560	7
	Necrosis	76	3	2189	13
	Vesicles	96	7	1444	6
	Bullae	64	14	457	10
	Erythema	55	72	76	3
Systemic	Coagulopathy (VICC/DIC)	1058	34	3134	35
	Systemic bleeding	242	10	2485	27
	Renal involvement	17	6	292	9
	Fever	19	12	157	3
	Neurologic	4	1	415	2
	ARDS	2	1	289	2

Abbreviations: ARDS, Acute Respiratory Distress Syndrome; DIC, Disseminated Intravascular Coagulation; VICC, Venom-Induced Consumptive Coagulopathy. Note: n/N (%) format throughout, with the number of contributing studies given for each item. Because denominators are variable-specific, findings that were not systematically assessed are necessarily underestimated relative to their true frequency; items documented by fewer than ten reports should be read as “reported in k studies (n = N)” rather than as population frequencies. Erythema, assessed in only three reports, illustrates this most clearly. All counts are at patient level.

## Data Availability

No new data were created in this study. All data discussed are contained within the cited publications; the complete extraction table underlying the frequencies reported in this review is provided as Supplementary Table S2. The original photographs reproduced in Figure 1 are the authors’ own and are published here for the first time.

## Supplementary Materials

The following supporting information can be downloaded at: https://www.mdpi.com/article/10.3390/jcm15218569/s1, Table S1: Complete electronic search strings and records retrieved; Table S2: Characteristics of the included studies, with species attribution, method of species identification, and the outcome domains each report documented; Table S3: Full texts assessed and not retained, with reasons; and patient-level exclusions applied within studies that were retained; Table S4: Synonymy of taxon names appearing in the included clinical literature, with the currently accepted combination; Figure S1: Flow diagram of study identification, screening, and selection for the clinical evidence base of this review. References [2,3,4,6,7,8,9,10,11,12,13,14,15,16,17,18,19,20,21,22,23,24,25,26,27,28,29,30,31,32,33,34,35,36,37,38,39,40,41,42,43,44,45,46,47,48,49,50,51,52,53,54,55,56,57,58,59,60,61,62,63,64,65,66,67,68,69,70,71,72,73,77,80,81,94,95,115] are cited in the Supplementary Materials.
