# Supplementary material for: The Clinical Phenotype of Green Pit Viper (Trimeresurus spp.) Envenomation: A Comprehensive Review
Source: J Clin Med. 2026 Nov 3;15(21):8569. doi: 10.3390/jcm15218569 (PMC13763668; doi:10.3390/jcm15218569)

Figure S1. Flow diagram of study identification, screening, and selection for the clinical evidence base of this review.

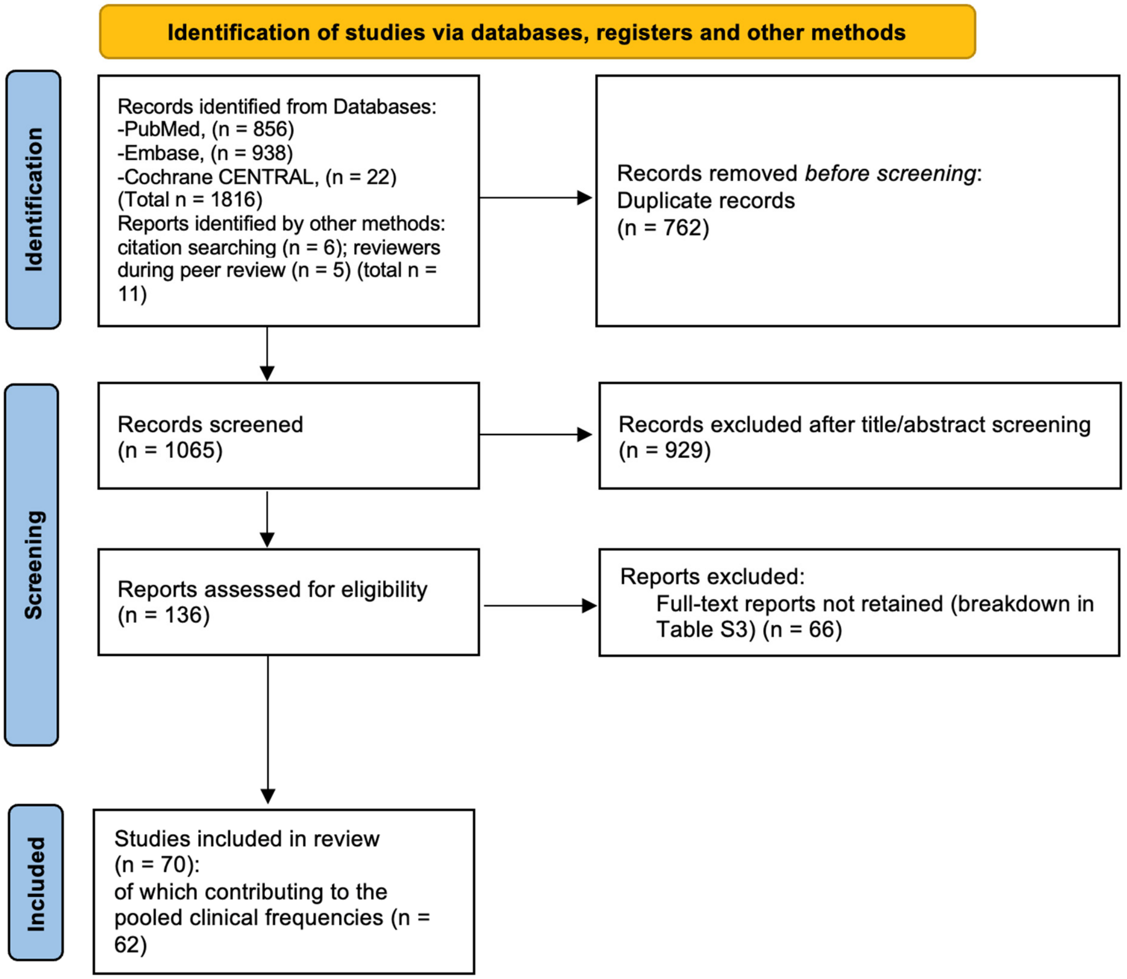

Supplement: Supplementary file 1 [file jcm-15-08569-s001.zip › Figure S1.pdf]
