# Supplementary material for: The Clinical Phenotype of Green Pit Viper (Trimeresurus spp.) Envenomation: A Comprehensive Review
Source: J Clin Med. 2026 Nov 3;15(21):8569. doi: 10.3390/jcm15218569 (PMC13763668; doi:10.3390/jcm15218569)
Supplement: Supplementary file 1 [file jcm-15-08569-s001.zip › Table S1.pdf]

Table S1. Complete electronic search strings and records retrieved. The species block and the clinical block were combined with AND; terms within each block were combined with OR. No language, date or publication-type filters were applied at the search stage.

Database	Interface / segment searched	Search string	Records retrieved
PubMed	All fields with field tags as shown; inception to 14 October 2026	("*Trimeresurus*"[MeSH Terms] OR *Trimeresurus*[tiab] OR *Cryptelytrops*[tiab] OR *Viridovipera*[tiab] OR *Parias*[tiab] OR *Popeia*[tiab] OR *Himalayophis*[tiab] OR *Sinovipera*[tiab] OR "green pit viper"[tiab] OR "green pit vipers"[tiab] OR "green pitviper"[tiab] OR "green pitvipers"[tiab] OR "bamboo viper"[tiab] OR "bamboo vipers"[tiab] OR "bamboo pit viper"[tiab] OR "white-lipped pit viper"[tiab] OR "white-lipped pit vipers"[tiab] OR "white-lipped tree viper"[tiab] OR "white-lipped green pit viper"[tiab] OR "mangrove pit viper"[tiab] OR "shore pit viper"[tiab] OR "island pit viper"[tiab]) AND (envenom*[tiab] OR snakebite*[tiab] OR "snake bite"[tiab] OR "snake bites"[tiab] OR bite[tiab] OR bites[tiab] OR bitten[tiab] OR antivenom*[tiab] OR antivenin*[tiab] OR venom*[tiab])	856
Embase	Emtree and free text (:ti,ab,kw); inception to 14 October 2026	('trimeresurus'/exp OR trimeresurus:ti,ab,kw OR cryptelytrops:ti,ab,kw OR viridovipera:ti,ab,kw OR parias:ti,ab,kw OR popeia:ti,ab,kw OR himalayophis:ti,ab,kw OR sinovipera:ti,ab,kw OR 'green pit viper':ti,ab,kw OR 'green pit vipers':ti,ab,kw OR 'green pitviper':ti,ab,kw OR 'green pitvipers':ti,ab,kw OR 'bamboo viper':ti,ab,kw OR 'bamboo vipers':ti,ab,kw OR 'bamboo pit viper':ti,ab,kw OR 'bamboo pit vipers':ti,ab,kw OR 'white-lipped pit viper':ti,ab,kw OR 'white-lipped pit vipers':ti,ab,kw OR 'white-lipped tree viper':ti,ab,kw OR 'white-lipped green pit viper':ti,ab,kw OR 'mangrove pit viper':ti,ab,kw OR 'shore pit viper':ti,ab,kw OR 'island pit viper':ti,ab,kw OR *albolabris*:ti,ab,kw OR *stejnegeri*:ti,ab,kw OR *erythrurus*:ti,ab,kw OR *purpureomaculatus*:ti,ab,kw OR *popeiorum*:ti,ab,kw OR *popeorum*:ti,ab,kw OR *flavomaculatus*:ti,ab,kw OR *sabahi*:ti,ab,kw OR *loong*:ti,ab,kw) AND ('snake bite'/exp OR envenom*:ti,ab,kw OR 'snake bite':ti,ab,kw OR 'snake bites':ti,ab,kw OR snakebite*:ti,ab,kw OR bite:ti,ab,kw OR bites:ti,ab,kw OR bitten:ti,ab,kw OR antivenom*:ti,ab,kw OR antivenin*:ti,ab,kw OR venom*:ti,ab,kw)	938
Cochrane CENTRAL	MeSH and free text (:ti,ab,kw); inception to 14 October 2026	#1 MeSH descriptor: [Crotalinae] explode all trees #2 MeSH descriptor: [Snake Bites] explode all trees #3 (*Trimeresurus* OR *Cryptelytrops* OR *Viridovipera* OR *Parias* OR *Popeia* OR *Himalayophis* OR *Sinovipera*):ti,ab,kw #4 ("green pit viper" OR "green pit vipers" OR "green pitviper" OR "green pitvipers" OR "bamboo viper" OR "bamboo vipers" OR "bamboo pit viper" OR "white-lipped pit viper" OR "white-lipped pit vipers" OR "white-lipped tree viper" OR "white-lipped green pit viper" OR "mangrove pit viper" OR "shore pit viper" OR "island pit viper" OR *albolabris* OR *stejnegeri* OR *erythrurus* OR *purpureomaculatus* OR *popeiorum* OR *popeorum* OR *flavomaculatus* OR *sabahi* OR *loong*):ti,ab,kw #5 #1 OR #3 OR #4 #6 (envenom* OR snakebite* OR "snake bite" OR "snake bites" OR bite OR bites OR bitten OR antivenom* OR antivenin* OR venom*):ti,ab,kw #7 #2 OR #6 #8 #5 AND #7	22 (22 Trials, 0 Reviews, 0 Protocols)
		Total retrieved from databases	1,816

All multi-word vernacular names are enclosed in quotation marks and tagged [tiab]. This is essential: without quotation marks, PubMed's automatic term mapping expands the component word "viper" to Crotalinae[MeSH Terms] exploded, which retrieves the entire subfamily and returns several thousand irrelevant records. Singular and plural forms of each vernacular phrase are given explicitly, and the clinical block uses truncation, because PubMed does not stem terms carried by a field tag: "bite"[tiab] does not retrieve "bites". Eleven further reports were identified by other methods - six through citation searching of the included reports and five by reviewers during peer review - and are shown in the corresponding arm of Figure S1; six of these name no *Trimeresurus* taxon in title, abstract or MeSH terms and are therefore not retrievable by any species-anchored search. Bare specific epithets were deliberately excluded from the species block: several (*gracilis*, *insularis*, *macrops*, *venustus*, *nebularis*, *sumatranus*, *hageni*) are shared with unrelated taxa or anatomical terms and retrieve large volumes of irrelevant records, while the species concerned are reliably captured by the genus and vernacular terms.
