# Supplementary material for: The Clinical Phenotype of Green Pit Viper (Trimeresurus spp.) Envenomation: A Comprehensive Review
Source: J Clin Med. 2026 Nov 3;15(21):8569. doi: 10.3390/jcm15218569 (PMC13763668; doi:10.3390/jcm15218569)
Supplement: Supplementary file 1 [file jcm-15-08569-s001.zip › Table S2.pdf]

Table S2. Characteristics of the included studies, with species attribution, method of species identification, and the outcome domains each report documented.

Study	Ref.	PMID / DOI	Year	Country	n	Species as reported	Identification	Cut.	Sys.	Site	Mgmt	Out.	Role / note
Pandey DP, Ghimire A, Shrestha BR	[42]	30737155	2019	Nepal	1	*T. albolabris*	not stated	Y	Y	Y	Y	Y	Included
Bhatt N, Singh A, Sharma SK	[7]	32228791	2020	Nepal	12	green pit viper	not stated	Y	–	Y	Y	Y	Included
Blessmann J, Khonesavanh C, Outhaithit P, et al.	[10]	20578499	2010	Laos	21	green pit viper	not stated	Y	Y	Y	Y	–	Included
Blessmann J, Nguyen TPN, Bui TPA, et al.	[9]	30448540	2018	Vietnam	98	green pit viper	not stated	–	–	–	Y	Y	Included
Blessmann J, Hanlodsomphou S, Santisouk B, et al.	[8]	36416013	2023	Laos	2	*Trimeresurus* spp.	not stated	–	Y	–	Y	Y	Included
Buranasin P	[12]	8362295	1993	Thailand	72	green pit viper	not stated	Y	Y	–	–	Y	Included
Chan K, Chung C	[14]	10.1177/102490790601300307	2006	Hong Kong	1	*T. albolabris*	not stated	Y	Y	Y	Y	Y	Included
Chan TY, Critchley JA	[17]	8067819	1994	Hong Kong	43	*T. albolabris*	not stated	Y	Y	Y	–	Y	Included
Chan T, Hung LK	[15]	20578498	2010	Hong Kong	1	*T. albolabris*	not stated	Y	–	Y	Y	Y	Included
Chan JC, Kwok MM, Cockram CS, et al.	[13]	8266156	1993	Hong Kong	21	*T. albolabris*	not stated	–	Y	–	–	Y	Included
Chan XY, Anthonysamy J, Sivaganabalan R, et al.	[18]	10.1016/j.toxicon.2024.108086	2024	Malaysia	25	*T. purpureomaculatus*	not stated	Y	Y	Y	Y	Y	Included
Chen YC, Chen MH, Yang CC, et al.	[19]	10.4269/ajtmh.2007.77.847	2007	Taiwan	3	*T. stejnegeri*	not stated	Y	Y	Y	Y	–	Included
Chen YW, Chen MH, Chen YC, et al.	[21]	10.4269/ajtmh.2009.80.28	2009	Taiwan	51	*T. stejnegeri*	not stated	Y	Y	Y	Y	Y	Included; the *V. stejnegeri* subgroup (51 of 200 patients) was correctly isolated from a two-species cohort, and the 41/10 upper-to-lower limb split matches Table 1 of the source article.
Chotenimitkhun R, Rojnuckarin P	[22]	18259959	2008	Thailand	243	*Trimeresurus albolabris* or *T. macrops*	not stated	Y	Y	–	Y	Y	Included

Nehaj F, Kubašková M, Mokáň M, et al.	[40]	30441986	2018	Not stated	1	*T. albolabris*	not stated	–	–	–	–	–	Included
Thang VV, Bao TQQ, Tuyen HD, et al.	[51]	32555599	2020	Vietnam	414	*Trimeresurus albolabris*	not stated	–	Y	–	Y	Y	Included
Vongphoumy I, Chanthilat P, Vilayvong P, et al.	[55]	10.1016/j.toxicon.2016.03.011	2016	Laos	26	*Trimeresurus albolabris*	not stated	–	–	–	–	–	Included
Ho CH, Mao YC, Tsai YD, et al.	[30]	10.4103/jmedsci.jmedsci_68_18	2019	Taiwan	9	*T. stejnegeri*	not stated	–	–	–	Y	Y	Included
Greene S, Galdamez LA, Tomasheski R	[27]	29102099	2017	USA	1	*Trimeresurus albolabris*	not stated	Y	Y	Y	Y	Y	Included
Sontichai W, Reungrongrat S, Narongchai P, et al.	[48]	25890858	2015	Thailand	1	*Trimeresurus albolabris*	not stated	Y	Y	Y	Y	Y	Included
Huang CY, Hung DZ, Chen WK	[32]	10.1016/S1726-4901(10)70117-9	2010	Taiwan	1	*Trimeresurus stejnegeri*	not stated	Y	Y	Y	Y	Y	Included
Hon KL, Lee KW, Cheung KL, et al.	[31]	19397540	2009	Hong Kong	1	*Trimeresurus albolabris*	not stated	Y	Y	–	Y	Y	Included
Thumtecho S, Tangtrongchitr T, Srisuma S, et al.	[4]	32801726	2020	Thailand	288	*Trimeresurus albolabris*	not stated	Y	Y	Y	Y	Y	Included
Ko JH, Chung WH	[34]	22884753	2013	Taiwan	1	*Trimeresurus stejnegeri*	not stated	–	–	Y	Y	Y	Included
Zheng Z, Yu Y, Wu Y, et al.	[59]	32576355	2020	China	48	*Trimeresurus stejnegeri*	not stated	–	Y	–	Y	Y	Included
Nuchprayoon I, Pongpan C, Sripaiboonkij N	[41]	18817605	2008	Thailand	43	*Trimeresurus albolabris*	not stated	Y	–	–	Y	Y	Included
Dumavibhat B	[26]	9347666	1997	Thailand	156	green pit viper	not stated	–	–	–	–	–	Included
Chan TY, Chan JC, Tomlinson B, et al.	[16]	7939957	1993	China	31	*Trimeresurus albolabris*	not stated	Y	–	–	–	–	Included
Sitprija V, Boonpucknavig V	[47]	912952	1977	Not stated	8	green pit viper	not stated	–	Y	–	–	–	Included
Bunlupunthaunas W	[11]	2637904	1989	Thailand	1	green pit viper	not stated	–	Y	–	Y	Y	Included
Yang JY, Hui H, Lee AC	[56]	17914147	2007	China	1	*Trimeresurus albolabris*	not stated	Y	Y	Y	Y	Y	Included

Thiansookon A, Rojnuckarin P	[53]	17996842	2008	Thailand	213	green viper	not stated	–	–	–	Y	Y	Included
Dumavibhat B, Visudhiphan S, Malasit P	[67]	2584906	1989	Thailand	2	green viper	not stated	–	Y	–	Y	Y	Included
Rojnuckarin P, Chanthawibun W, Noiphrom J, et al.	[44]	16466758	2006	Thailand	28	*Trimeresurus albolabris* or *T. macrops*	not stated	Y	–	Y	Y	–	Included
Cockram CS, Chan JC, Chow KY	[23]	2325197	1990	Hong Kong	–	*Trimeresurus albolabris*	not stated	–	–	–	–	Y	Cited for mortality outcome only (one probable *T. albolabris* fatality among 242 all-species snakebite admissions); *Trimeresurus*-specific sample size not separable from the abstract, so the report contributes to the mortality numerator but not to any pooled denominator.
Lee JT	[35]	https://www.scopus.com/pages/publications/0033379949	1999	Taiwan	1	*T. stejnegeri*	not stated	–	–	–	Y	–	Included
Hutton RA, Looareesuwan S, Ho M, et al.	[2]	2096527	1990	Thailand	29	24 by *Trimeresurus albolabris* and 5 by *T. macrops*	not stated	Y	Y	–	Y	Y	Included
Zeng L, Liang Q, Liang Z, et al.	[58]	35045831	2022	China	123	*T. albolabris, T. stejnegeri*	not stated	Y	Y	–	Y	Y	Included
Sirur FM, Lath V, A S C, et al.	[46]	34865965	2022	India	1	*Trimeresurus malabaricus*	not stated	–	–	–	–	–	Excluded from counts (reassigned to *Craspedocephalus*); retained in the 70-study bibliography as a historical *Trimeresurus* report, but excluded from the 4,720 species and clinical counts (see Table S3 and Table S4).
Tongpoo A, Niparuck P, Sriapha C, et al.	[54]	35154756	2020	Thailand	24	*Trimeresurus albolabris*, *Trimeresurus macrops*	not stated	Y	–	Y	Y	Y	Included
Shankar T, Arora P, Jose JR, et al.	[45]	38782420	2024	India	1	*Trimeresurus albolabris*	not stated	–	Y	–	Y	Y	Included

Chen YH, Mao YC, Liu PY, et al.	[20]	35987354	2022	Taiwan	1	*Trimeresurus stejnegeri*	per-patient table	Y	Y	Y	Y	Y	Included
Qamruddin RM, Safferi RS, Mohamed Ismail Z, et al.	[43]	37585486	2023	Malaysia	208 (of 523)	pit viper	expert	–	–	Y	Y	Y	Species counts only; Table 2 of the source resolves to 208 eligible *Trimeresurus* patients (*T. purpureomaculatus* 124, *T. sabahi fucatus* 26, *T. hageni* 24, *T. sumatranus* 15, *T. nebularis* 13, *T. sabahi sabahi* 4, *T. venustus* 1, and one imported *T. flavomaculatus*), 34 patients bitten by taxa now placed in *Craspedocephalus* (*T. borneensis* 28, *T. wiroti* 6), and 281 bitten by other genera (*Tropidolaemus* subannulatus 121, *Tropidolaemus wagleri* 84, *Calloselasma rhodostoma* 58, *Ovophis* convictus 17, *Garthius* chaseni 1). The clinical data are not disaggregable by genus, so the report does not contribute to the pooled frequencies.
Lu HY, Mao YC, Liu PY, et al.	[38]	36945006	2023	China	63	*Trimeresurus stejnegeri*	not stated	Y	Y	Y	Y	–	Included
Dong D, Deng Z, Yan Z, et al.	[25]	33117427	2020	China	3	*Trimeresurus stejnegeri*	not stated	Y	–	–	Y	–	Included; primarily a proteomic study, but reports three genuine *T. stejnegeri* patients.
Yoshimura K, Hossain M, Tojo B, et al.	[57]	37782628	2023	Bangladesh	4	*Trimeresurus albolabris*	not stated	–	–	–	–	Y	Included
Gupta A, Bhandari S, Anand A, et al.	[28]	36299668	2022	Nepal	1	green pit viper	not stated	Y	Y	Y	Y	Y	Included
Ho CH, Ismail AK, Liu SH, et al.	[29]	33605805	2021	Taiwan	5	Crotalinae snakes	per-patient table	Y	–	–	Y	–	Species counts only; the per-patient table identifies patients 1–5 as *T. stejnegeri* and 6–17 as *P. mucrosquamatus*, so only the five *Trimeresurus* cases are eligible and the report

													does not contribute to the pooled frequencies.
Liang M, Liang P, Luo W, et al.	[37]	35307067	2022	China	265	*Trimeresurus* spp.	not stated	–	–	–	–	–	Species counts only; abstract only. The 265 *Trimeresurus* patients are 27.84% of the 952 patients with venomous snakebites in the series. Local symptoms are described qualitatively, without patient counts, so the report does not contribute to the pooled frequencies.
Thalgaspitiya S, Isbister G, Ukuwela K, et al.	[50]	32891665	2020	Sri Lanka	1	*Trimeresurus trigonocephalus*	not stated	–	–	–	–	–	Excluded from counts (reassigned to *Craspedocephalus*); retained in the 70-study bibliography as a historical *Trimeresurus* report, but excluded from the 4,720 species and clinical counts (see Table S3 and Table S4).
Thein MM, Rogers CA, White J, et al.	[52]	34562496	2021	Myanmar	22	*Trimeresurus albolabris* (21) and *Trimeresurus erythrurus* (1)	not stated	Y	Y	Y	Y	Y	Included; 21 *T. albolabris* bites confirmed by expert identification of retained snakes (the 22nd retained snake was *Ahaetulla prasina*, a non-venomous colubrid, and is not counted), plus one separately reported, snake-identified *T. erythrurus* case with acute kidney injury from Yangon in the same article.
Othong R, Keeratipornruedee P	[68]	31913729	2020	Thailand	320	*Trimeresurus albolabris* and *Trimeresurus macrops*	not stated	Y	Y	Y	Y	Y	Included; Vajira Hospital, 2008–2015. The 2011–2015 portion of this window is shared with the larger Othong 2022 cohort (approximately 200 patients); the overlap is declared in Section 2 and the figures are reported as patient episodes rather than unique patients.

Thakur S, Giri S, Lalremsanga HT, et al.	[49]	38531479	2024	India	62	green pit vipers	not stated	Y	Y	–	Y	Y	Included; review-format article containing the authors' own primary data (62 patients, Demow Community Health Centre, September 2022 to December 2023). The enrolment window is consecutive with, and does not overlap, the Kakati 2023 cohort from the same centre.
Luo Y, Zhang J, Zhai C, et al.	[39]	33198872	2020	China	120	*Trimeresurus stejnegeri*	not stated	Y	Y	–	Y	Y	Included
Dang XT, Nguyen TX, Nguyen HTT, et al.	[24]	35023369	2022	Vietnam	33 (of 41)	*Trimeresurus albolabris* (22), *Trimeresurus mucrosquamatus* (7), *Trimeresurus cornutus* (1), *Deinagkistrodon* acutus (1), undefined (10)	not stated	–	Y	–	Y	Y	Included; of 41 patients, only the 22 *T. albolabris* are entered in the species counts. Clinical outcomes are reported for all 41 together and are entered as reported (33 of 41, excluding the 8 *Protobothrops* cases), so they are not specific to *Trimeresurus*: the remainder comprises 1 *Deinagkistrodon* acutus and 10 snakes recorded as undefined.
Othong R, Eurcherdkul T, Chantawatsharakorn P	[3]	36548767	2022	Thailand	760	*Trimeresurus albolabris* or *Trimeresurus macrops*	not stated	Y	Y	Y	Y	Y	Included; Vajira and Taksin Hospitals, 2011–2021. This enrolment window contains that of Othong & Keeratipornruedee 2020 and that of the nested Kriengkrairut cohort; as the larger report it is the one retained.
Kakati H, Giri S, Patra A, et al.	[33]	37257518	2023	India	92	*Trimeresurus erythrurus* (green pit viper), *Trimeresurus salazar*	not stated	Y	Y	–	Y	Y	Included
Li Q, Huang Y, Zhang Y, et al.	[36]	38668745	2024	China	68 (of 69)	*Trimeresurus albolabris* (35), *Protobothrops mucrosquamatus* (1), *Naja atra* (7), *Daboia russelii siamensis* (2),	not stated	Y	Y	Y	Y	Y	Included; of 69 children, only the 35 *T. albolabris* are entered in the species counts. Clinical outcomes are reported for all 69 children together and are

						Bungarus multicinctus (2), unidentified (22)						entered as reported (68 of 69, excluding the single *Protobothrops* case), so they are not specific to *Trimeresurus*: 11 children were bitten by *Naja*, *Daboia* or *Bungarus* and 22 by unidentified snakes.
Chiang LC, Tsai WJ, Liu PY, et al.	[62]	32983233	2020	Taiwan	185	*Trimeresurus stejnegeri stejnegeri*	not stated	Y	Y	Y	Y	Y	Included; one death (woman aged 71, gastrointestinal bleeding and shock, 18 h after the bite); necrosis 13, ecchymosis 53, coagulopathy 15, renal impairment 3; 6 patients underwent surgery, with 11 procedures in total (6 debridements, 1 fasciotomy, 2 grafts).
Hung YL, Chen YC, Tsai TS, et al.	[65]	38109772	2024	Taiwan	254	*Trimeresurus stejnegeri*	not stated	Y	–	Y	Y	Y	Included; wound infection in 65 of 254 patients (26%); 14 patients underwent surgery, with fasciotomy in 12; all patients received antivenom, which was an enrolment criterion.
Chan NTJ, Lam PKR, Chan CK, et al.	[60]	39427850	2024	Hong Kong	82	*Trimeresurus albolabris*	not stated	Y	Y	–	Y	Y	Cited for specific data; the cohort was selected on coagulopathy of at least grade 2 (mSSS), so entering it into the pooled frequencies would give 82 of 82 (100%) by construction. Used only for its door-to-antivenom findings.
Chen MH, Hu HF, Wu SH, et al.	[66]	39466297	2025	Taiwan	2	*Trimeresurus gracilis*	not stated	Y	Y	Y	Y	Y	Included; 2 patients and 3 events; necrosis, compartment syndrome, and coagulopathy without spontaneous bleeding; a paraspecific antivenom controlled the local toxicity.
Niruntarai S, Hengtrakul S, Othong R	[64]	41838663 / 10.1371/journal.pntd.0014121	2026	Thailand	39	green pit viper (*Trimeresurus* spp.)	Clinical/vernacular	–	Y	–	Y	Y	Included; prospective, Vajira Hospital Sep 2022–Nov 2023 (no overlap with

													[3] or [68]); provides 20WBCT–fibrinogen thresholds.
Kaphle P, Gupta P, Shrestha S, et al.	[63]	42339145 / 10.1002/ccr3.73002	2026	Nepal	1	presumed green pit viper (*Trimeresurus* spp.)	Clinical/vernacular	Y	Y	–	Y	Y	Included; second reported case of microangiopathic hemolytic anemia in this genus.
Yan S, Fu W, Yan Z, et al.	[61]	42044109 / 10.1371/journal.pntd.0013247	2026	China	40,817 total; *T. stejnegeri* 11.40%	*Trimeresurus stejnegeri*; 56.36% unidentified	Not stated	–	–	–	Y	Y	Cited for specific data; cross-sectional survey, manifestations reported for the whole all-species cohort and not separable by species, so not pooled.
Mitrakul C, Impun C	[69]	4540743 / 10.1177/000992287301200409	1973	Thailand	13 (of 20)	'green pit viper' - *T. erythrurus* and *T. popeorum*, not separable	Clinical/vernacular	Y	Y	–	–	–	Included; paediatric series, Chulalongkorn Hospital; only the 13 with an identified green pit viper are eligible, the other 7 snakes were unidentified; earliest study in the series.
Khurshid T, Alam MS, Ely NF, et al.	[70]	10.3329/jom.v26i1.78993	2025	Bangladesh	112	'Green Pit Viper' - genus only	Snake brought to hospital	Y	Y	–	Y	Y	Included; all snakes were brought in and identified, yet recorded only by vernacular name - see Section 4.4.
Suen PY	[71]	10.1177/102490791702400205	2017	Hong Kong	63	*Trimeresurus albolabris*	Clinical/vernacular	Y	Y	–	Y	Y	Included; retrospective cohort 2001–2015, regional hospital; enrolment window overlaps 2012–2015 with [60], which does not contribute to the pooled frequencies.
Priandhini SA, Insyirach NI, Atsira OP, et al.	[72]	10.5339/jemtac.2025.9	2025	Indonesia	1	*Trimeresurus insularis*	Photographic/expert	Y	Y	Y	Y	Y	Included; first *T. insularis* case in the series; tetanus on day 8.
Saengnoi T, Chantrathammachart P, Puavilai T, et al.	[73]	http://www.jmatonline.com/PDF/1213-21_10005.pdf	2019	Thailand	21 (of 30)	'green pit viper' - genus only	Clinical/vernacular	Y	Y	–	Y	–	Included; prospective cohort, Sawanpracharak Hospital 2016–2017; fully disaggregated from the 9 *Daboia russelii* cases in every table.

Y = the domain is documented in the report; – = not documented (or not entered into pooled denominators). Cut. = cutaneous; Sys. = systemic; Site = bite site; Mgmt = management; Out. = outcome. "Role" indicates whether a report is one of the 62 studies contributing to the pooled clinical frequencies (Included), or one of the eight non-pooled studies: cited only for species counts (Species counts

only, n = 3), cited for specific data without entering the pooled frequencies (Cited for specific data, n = 2), contributing a fatality to the mortality numerator without an extractable denominator (Cited for mortality outcome only, n = 1), or retained in the bibliography as a historical *Trimeresurus* report but excluded from all counts following reassignment to *Craspedocephalus* (Excluded from counts, n = 2). Identification = method by which the offending snake was identified, where stated. Ref. = number of the corresponding entry in the reference list of the main text; bracketed numbers in the Role/note column refer to that same list.
