# Supplementary material for: The Clinical Phenotype of Green Pit Viper (Trimeresurus spp.) Envenomation: A Comprehensive Review
Source: J Clin Med. 2026 Nov 3;15(21):8569. doi: 10.3390/jcm15218569 (PMC13763668; doi:10.3390/jcm15218569)
Supplement: Supplementary file 1 [file jcm-15-08569-s001.zip › Table S3.pdf]

Table S3. Full texts assessed and not retained, with reasons; and patient-level exclusions applied within studies that were retained.

Category / study	n	Stage	Reason
Reviews, epidemiological summaries or toxin-focused studies without original patient data	27	Original search + update	Does not meet the inclusion criterion requiring original human clinical data
Jalink MB et al., Toxin Reviews 2024 [6] (ocular sequelae)	1 (of the 27)	Reassessed during revision	Narrative review; the figure of 42 originally extracted was the number of case reports reviewed, not patients (65 cases, all previously published)
Conference abstracts and preprints	13	Updated search	Do not permit extraction of the data tabulated in Tables 1 and 2; in several instances the same work is already included as a full publication. Retrieved almost exclusively by the corrected Embase search.
Animal or in vitro studies	8	Original search + update	No human clinical data
Full texts that could not be accessed	4	Original search	Not retrievable through institutional or interlibrary access
Taxonomic reclassification outside the genus	6	Original search	*Trimeresurus flavoviridis* to *Protobothrops flavoviridis*; *Trimeresurus wagleri* to *Tropidolaemus wagleri*
Veterinary studies	2	Original search	Non-human envenomation
Duplicate cohort	1	Original search	Cases already reported in another included source
Nested cohort: Kriengkrairut S & Othong R, Emerg Med Australas 2021 [115]	1	Identified by reviewer	Vajira Hospital 2015-2019; enrolment window falls entirely within that of Othong et al. 2022 [3] (Vajira and Taksin, 2011-2021). Cited for its infection data but not added to the patient totals
Cohorts pooling *Trimeresurus* and *Protobothrops* without disaggregation	2	Reassessed during revision	Lin CC et al., Toxins 2020 [95] (n = 726; enrolled by diagnostic code or by receipt of a bivalent antivenom covering both genera); Chen YC et al., J Formos Med Assoc 2007 [94] (n = 179; 136 *Protobothrops mucrosquamatus*, outcomes stratified by treatment group rather than species)
Species belonging to *Craspedocephalus*	4 taxa, 36 patients	Patient-level (study retained)	*Trimeresurus malabaricus*, *T. trigonocephalus*, *T. borneensis* and *T. wiroti* are currently placed in *Craspedocephalus*
Non-*Trimeresurus* genera within an aggregate 'pit viper' series	281 patients	Patient-level (study retained)	Qamruddin RM et al. 2023 [43]: *Tropidolaemus subannulatus* 121, *Tropidolaemus wagleri* 84, *Calloselasma rhodostoma* 58, *Ovophis convictus* 17, *Garthius chaseni* 1
Genus and geographic region not relevant	1	Updated search	Report of envenomation by *Bothrops bilineatus*, a Neotropical species also known vernacularly as a green pit viper
Behavioral ecology	1	Updated search	No clinical content

Categories are listed in the same order and wording used in Section 2 and in Figure S1. The study-level categories (those with a count in the “n” column expressed as a number of reports) sum to the 66 full texts not retained. The final two rows are not study-level exclusions: they record patients removed from the pooled counts within reports that were otherwise retained, and they are therefore excluded from that total. Bracketed numbers refer to the reference list of the main text.
