# Supplementary material for: The Clinical Phenotype of Green Pit Viper (Trimeresurus spp.) Envenomation: A Comprehensive Review
Source: J Clin Med. 2026 Nov 3;15(21):8569. doi: 10.3390/jcm15218569 (PMC13763668; doi:10.3390/jcm15218569)
Supplement: Supplementary file 1 [file jcm-15-08569-s001.zip › Table S4.pdf]

**Table S4.** Synonymy of taxon names appearing in the included clinical literature, with the currently accepted combination.

| Name as used in the source literature | Currently accepted combination | Authority | Note |
|---|---|---|---|
| *Trimeresurus albolabris* | *Trimeresurus albolabris* | Gray, 1842 | Valid; complex under revision |
| *Cryptelytrops albolabris* | *Trimeresurus albolabris* | Gray, 1842 | *Cryptelytrops* treated as a synonym or subgenus of *Trimeresurus* |
| *Trimeresurus macrops* | *Trimeresurus macrops* | Kramer, 1977 | Valid species; formerly treated as a subspecies of *T. albolabris* |
| *Viridovipera stejnegeri* | *Trimeresurus stejnegeri* | Schmidt, 1925 | *Viridovipera* treated as a subgenus of *Trimeresurus* |
| *Trimeresurus mucrosquamatus* | *Protobothrops mucrosquamatus* | (Cantor, 1839) | Reassigned to *Protobothrops*. Excluded from this review |
| *Trimeresurus cornutus* | *Protobothrops cornutus* | (Smith, 1930) | Reassigned to *Protobothrops*. Excluded from this review |
| *Trimeresurus flavoviridis* | *Protobothrops flavoviridis* | (Hallowell, 1861) | Reassigned to *Protobothrops*. Excluded at the search stage |
| *Trimeresurus wagleri* | *Tropidolaemus wagleri* | (Boie, 1827) | Reassigned to *Tropidolaemus*. Excluded at the search stage |
| *Trimeresurus malabaricus* | *Craspedocephalus malabaricus* | (Jerdon, 1854) | Reassigned to *Craspedocephalus*. Excluded from this review |
| *Trimeresurus trigonocephalus* | *Craspedocephalus trigonocephalus* | (Donndorff, 1798) | Reassigned to *Craspedocephalus*. Excluded from this review |
| *Trimeresurus borneensis* | *Craspedocephalus borneensis* | (Peters, 1872) | Reassigned to *Craspedocephalus*. Excluded from this review |
| *Trimeresurus wiroti* | *Craspedocephalus wiroti* | (Trutnau, 1981) | Reassigned to *Craspedocephalus*. Excluded from this review |
| *Trimeresurus puniceus* | *Craspedocephalus puniceus* | (Boie, 1827) | Reassigned to *Craspedocephalus*; cited only for venom proteomic data |
| *Trimeresurus liqibini* | *Trimeresurus loong* | Xu, Deng, Zhang, Nguyen, Weng, Poyarkov, Vogel, Sun, Liao & Peng, 2025 [77] | Synonymised with *T. loong* [81]; still listed separately in the Reptile Database at the access date |
| *Parias*, *Popeia*, *Himalayophis*, *Sinovipera* | *Trimeresurus* | - | Treated as subgenera within a broad concept of *Trimeresurus* |

Nomenclature follows the Reptile Database [80] (accessed 13 October 2026), which listed 60 valid species of *Trimeresurus* at that date. Authorities are reproduced from the individual Reptile Database species accounts. Parentheses indicate that the species was described in a genus other than the one in which it is currently placed; their absence indicates that the original and current generic combinations agree. Bracketed numbers refer to the reference list of the main text.

**Valid species of *Trimeresurus* at the access date (n = 60)**

| | | | |
|---|---|---|---|
| *Trimeresurus albolabris* | *Trimeresurus* andersonii | *Trimeresurus* arunachalensis | *Trimeresurus* ayeyarwadyensis |
| *Trimeresurus* calamitas | *Trimeresurus* cantori | *Trimeresurus* cardamomensis | *Trimeresurus* caudornatus |
| *Trimeresurus* ciliaris | *Trimeresurus* cryptographicus | *Trimeresurus* cyanolabris | *Trimeresurus* davidi |
| *Trimeresurus* erythrochloris | *Trimeresurus erythrurus* | *Trimeresurus* fasciatus | *Trimeresurus flavomaculatus* |
| *Trimeresurus gracilis* | *Trimeresurus* gumprechti | *Trimeresurus* gunaleni | *Trimeresurus hageni* |
| *Trimeresurus* hongheensis | *Trimeresurus* honsonensis | *Trimeresurus insularis* | *Trimeresurus* kanburiensis |
| *Trimeresurus* kirscheyi | *Trimeresurus* kraensis | *Trimeresurus* kuiburi | *Trimeresurus* labialis |
| *Trimeresurus* lanna | *Trimeresurus* lii | *Trimeresurus liqibini* | *Trimeresurus loong* |
| *Trimeresurus macrops* | *Trimeresurus* malcolmi | *Trimeresurus* mayaae | *Trimeresurus* mcgregori |
| *Trimeresurus* medoensis | *Trimeresurus* mutabilis | *Trimeresurus nebularis* | *Trimeresurus* nujiang |
| *Trimeresurus* phuketensis | *Trimeresurus popeiorum* | *Trimeresurus* pretiosus | *Trimeresurus purpureomaculatus* |
| *Trimeresurus* rubeus | *Trimeresurus sabahi* | *Trimeresurus salazar* | *Trimeresurus* schultzei |

| | | | |
|---|---|---|---|
| *Trimeresurus* septentrionalis | *Trimeresurus* sichuanensis | *Trimeresurus stejnegeri* | *Trimeresurus sumatranus* |
| *Trimeresurus* tenasserimensis | *Trimeresurus* tibetanus | *Trimeresurus truongsonensis* | *Trimeresurus* uetzi |
| *Trimeresurus venustus* | *Trimeresurus* vogeli | *Trimeresurus* whitteni | *Trimeresurus* yunnanensis |
